# Assessing depression, anxiety, stress, and occupational decision regret levels among resident physicians working at Ankara University Faculty of Medicine Hospital

**DOI:** 10.55730/1300-0144.5875

**Published:** 2024-07-25

**Authors:** Emrah EMİRAL, Baris ORS, Nergis CANTÜRK

**Affiliations:** 1 Department of Forensic Medicine, Faculty of Medicine, Ankara University, Ankara, Turkiye; 2 Department of Emergency Health Services, Ankara Provincial Health Directorate, Ankara, Turkiye; 3 Department of Criminalistics, Institute of Forensic Sciences, Ankara University, Ankara, Turkiye

**Keywords:** DASS-21, occupational decision regret, physicians, faculty of medicine

## Abstract

**Background/aim:**

Physicians work under high levels of stress due to factors such as excessive workload, emotional factors, and economic variables. This leads to various health problems such as depression, burnout, fatigue, and hopelessness, resulting in decreased interest in a medical career and an increase in career choice regret.

**Materials and methods:**

The study included 300 volunteer resident physicians from Ankara University Medical Faculty Hospital. The data for the research were collected using a survey form prepared by reviewing the literature. The survey consisted of three parts, which questioned the physicians’ sociodemographic characteristics and professional choices, including the Depression Anxiety Stress Scale-Short Form (DASS-21) items and the Decision Regret Scale.

**Results:**

Of the physicians, 216 (72.0%) chose the medical faculty due to personal preference. The percentage of those who were not regretful about their career choice was 14.3% (n = 43). Those not regretful about their career choice had fewer years in the profession than the others. According to the categorical assessment of the DASS-21, 73.7% (n = 221) of the physicians had depressive symptoms ranging from mild to severe, 78.7% (n = 236) had anxiety symptoms ranging from mild to severe, and 57.7% (n = 173) had stress symptoms ranging from mild to severe.

**Conclusion:**

Mental health problems such as depression, anxiety, and stress were common among the resident physicians independent of their sociodemographic characteristics, and this was also associated with the level of career regret. Improving working conditions and personal benefits, addressing economic and other issues for physicians, ensuring their well-being, preventing the development of mental health problems, and early screening and rehabilitation for those affected not only have personal benefits but also contribute positively to job satisfaction, strengthen the patient–physician relationship, and have a significant impact on healthcare services.

## 1. Introduction

Career choice is influenced by factors such as the work environment, stress, income, and social opportunities, potentially affecting career satisfaction [1]. The medical profession is preferred by families and young individuals due to the perception that it is an idealistic, prestigious, and well-paying occupation aimed at preserving human health [2]. Choosing a career in medicine is a complex personal decision influenced by numerous factors. Career choices are influenced by both the inclinations of graduates before entering medical school and the gains during medical school education [3].

The medical profession plays a significant role in preserving, maintaining, and improving the health of society. However, while fulfilling these responsibilities, physicians can encounter various stress factors and may need to cope with multiple challenges. Physicians often experience stress due to factors such as excessive workload and emotional factors [4]. This can lead to various health issues, including depression, burnout, fatigue, hopelessness, self-care neglect, suicide, and substance abuse [5,6]. A systematic review showed that in the United Kingdom, 31%–54% of physicians experienced burnout, and 17%–52% had a psychiatric illness [7]. These mental health conditions are particularly prevalent in the early years of physicians’ careers [8].

Additionally, the accompanying stress significantly affects physicians’ cognitive function and reduces their clinical decision-making abilities [9]. It has been shown that preventing stress reduces burnout and potential medical errors [10]. A study found that physicians’ professional satisfaction is positively related to patient satisfaction [11].

Regret is often associated with the high-stress nature of the medical field and its many negative effects on health [12]. Previous studies have shown that depressive symptoms and higher levels of burnout are associated with decreased interest in a medical career and increased career choice regret [13,14]. Based on this, the current study aimed to determine the levels of depression, anxiety, stress, and occupational decision regret among resident physicians working at Ankara University Faculty of Medicine Hospital.

## 2. Materials and methods

Ethical approval for the study was obtained from the Ankara University Faculty of Medicine Human Research Ethics Committee, with the decision dated 26.09.2022 and numbered I08-526-22.

### 2.1. Preparation of surveys

The data for the research were collected by the researchers using a survey form prepared through a literature review. The survey form consists of three sections. The first section includes items that inquire about the sociodemographic characteristics of the physicians (such as age, sex, etc.) and their career choices. The second section contains propositions from the Depression, Anxiety Stress Scale-Short Form (DASS-21). Antony et al. [15] adapted this 21-item version of the scale from the original DASS-42, which was developed by Lovibond and Lovibond [16], and consists of 42 questions.

The Turkish validity and reliability study of the DASS-21 was conducted by Yılmaz et al. [17]. The DASS-21 is a self-report scale with a 4-point Likert-type response format that assesses anxiety, depression, and stress dimensions, each consisting of 7 items, in clinical and nonclinical samples. Responses to the scale are scored as follows: did not apply to me at all = 0 points; applied to me to some degree, or some of the time = 1 point; applied to me to a considerable degree or a good part of time = 2 points; and applied to me very much or most of the time = 3 points. Scores for each sub-dimension can range from 0 to 21, with higher scores indicating higher levels of depression, anxiety, or stress.

The third section of the survey form contains items from the Decision Regret Scale (DRS). The DRS is a measurement tool consisting of 5 items and a single dimension developed by Brehaut et al. [18]. The Turkish validity and reliability study of the scale was conducted by Erdurcan and Kırdök [19]. When developing the Turkish version of the scale, considerations were made to prevent misunderstandings in practice, ensure objectivity in the application and evaluation of the scale, and facilitate score calculations. Therefore, in the Turkish version, the scale is arranged on a 5-point Likert scale ranging from 0 = strongly disagree to 4 = strongly agree. As a result, in the Turkish version, items 1, 3, and 5 are reverse-coded. When scoring the scale, after reversing the scores of the three items, the scores are summed to obtain a total score. This score is then multiplied by five to obtain a value between 0 and 100. An increase in the calculated score between 0 and 100 indicates an increase in decision regret. If the score obtained from the scale is between 0 and 24, it can be said that the individual is ‘not regretful at all about the decision’. A score between 25 and 49 indicates ‘slightly regretful about the decision’, between 50 and 74 indicates ‘regretful due to the decision’, and between 75 and 100 indicates ‘very regretful due to the decision’.

### 2.2. Completion of the surveys by the resident physicians

Resident physicians in hospital wards and outpatient clinics were visited on designated days and hours. The subject and purpose of the study were explained to them. Those who agreed to participate in the study were provided with survey forms to be filled out by themselves. After 48 h, the same physicians were revisited, and the survey forms were collected.

### 2.3. Statistical analysis

Approximately 1200 resident physicians work at Ankara University Faculty of Medicine Hospital. In the study, the frequency of regretting their career choice among physicians was assumed to be 50%, with a 5% margin of error and a 95% confidence interval, resulting in a calculated sample size of 292.

The study data were analyzed using IBM SPSS Statistics for Windows 23.0 (IBM Corp., Armonk, NY, USA). Descriptive information for the study group was presented in terms of numbers, percentages, and the mean ± standard deviation (SD). The normality of the data was assessed using the Shapiro–Wilk test. The Mann–Whitney U/Kruskal–Wallis tests and Spearman correlation analysis were used in the univariate analysis of the data.

## 3. Results

Of 1200 resident physicians, 300 (25%) voluntarily participated in the study. Among these, 176 (58.7%) were female and 124 (41.3%) were male. Their ages ranged from 24 to 40 years, with a mean of 27.0 ± 1.9 and a median of 27. Moreover, 61 (20.3%) participants were married, and 216 (72.0%) had chosen the medical faculty due to personal preference. The physicians’ DRS scores ranged from 0.0 to 100.0, with a mean of 46.4 ± 28.2 and a median of 40.0. According to the categorical evaluation, 14.3% (n = 43) physicians were not regretful about their career choice.

No relationship was found between the physicians’ DRS scores and their sociodemographic and professional characteristics (p > 0.05 for both). These data are presented in Table 1.

Physicians who were not regretful about their career choice had fewer years in the profession compared to those who were slightly regretful and regretful about their career choice. No relationship was found between the levels of career decision regret and age. A comparison of the physicians’ age and number of years in the profession with their levels of career decision regret is provided in Table 2.

No relationship was found between the physicians’ DASS-21 scores and their sociodemographic and professional characteristics (p > 0.05 for both). These data are presented in Table 3.

According to the categorical assessment of the DASS-21, 73.7% of the physicians (n = 221) had mild to severe depressive symptoms, 78.7% (n = 236) had mild to severe anxiety symptoms, and 57.7% (n = 173) had mild to severe stress symptoms. The physicians’ DASS-21 scores are presented in Table 4, and the categorical assessment is provided in Table 5.

There was a strong to moderate positive correlation between the physicians’ DASS-21 and DRS scores. These data are presented in Table 6.

## 4. Discussion

The format and processes of medical training can vary among countries, but it is one of the longest and most challenging educational processes worldwide [3]. In Türkiye, to become a physician, one must first gain admission to medical school and then complete the country’s longest and most demanding undergraduate education, lasting six years. After that has been completed, one can start working as a general practitioner without needing an additional examination [20]. It is one of the rare professions in which, after completing undergraduate education, individuals can start their careers directly without undergoing another exam, with guaranteed employment. This can have a significant impact on both families and students. In the current study, about three-quarters of the physicians stated that they chose to attend medical school primarily for being beneficial to other people, followed closely by having job security. Different research conducted in Türkiye has also shown that most students choose the medical field as their first preference for reasons such as wanting to be a helpful member of society, and experiencing inner satisfaction [21,22]. In a study by Heikkila et al. conducted in Finland, where in they examined the reasons for choosing a medical career at 10-year intervals, the most common, ranging from 72% to 82% in each period, was dealing with people. In the same study, having a prestigious profession remained high among the reasons for choosing medical school. In contrast, the percentage of those stating for a good salary was shown to decline [23]. Similarly, in a study conducted in Ireland, the probability of being able to help others was also cited as the most common reason for choosing a medical career [24]. Despite the evident changes in the healthcare system and society, the current findings indicate that the most important factor in choosing the medical profession is still the intrinsic satisfaction with the profession’s content and the ability to help others.

In Türkiye, after completing their university education, physicians must take the Medical Specialization Examination (TUS) to choose their medical specialization. The duration of specialization typically ranges from three to six years under normal circumstances, and successful completion of a thesis and science exams is required at the end of this process [20]. The choice of specialization is a complex process influenced by various factors. Graduation year, personal experiences during the student years, personal life conditions, areas of interest, and the characteristics of the chosen specialization field (quality of training, duration, the possibility of subspecialty) and post-specialization working conditions are examples of these factors [25,26]. In the present study, just like the choice of the medical profession, interest in the field was the primary reason for choosing a specialization, followed by achieving the required score. In a study conducted in the United Kingdom, while analyzing specialization preference reasons in terms of age cohorts, chosen fields, ethnic background, and sex, the most common factor was interest in the field, followed by professional experiences gained. However, for men, the third was assessment of their skills, and for women, it was working conditions and hours [27]. In a study conducted in Sweden by Van der Horst et al., the three most influential reasons for specialization choices were the opportunity to treat complex diseases, a wide spectrum of diseases, and opportunities for communication with patients. The least mentioned factors in the same study were income level, not requiring a change of residence, and not working in the emergency department [26]. Another study conducted in Türkiye showed that the most important individual factors in choosing a specialization were suitability for one’s personality/abilities. Meanwhile, the most important systemic/professional factors were the TUS score and fewer on-call duties [28]. In a study that examined the changes in specialization preference scores over the years, it was observed that the placement scores for specialization fields such as pediatrics, cardiology, and pulmonology, which have demanding working conditions during both the residency and post-specialization periods, decreased over the years. This indicates that these fields were preferred less over time [20]. The medical profession is associated with high levels of professional stress, burnout, and emotional problems, alongside both individual and professional satisfaction. Research has shown that healthcare professionals, especially resident doctors and faculty members, are prone to having mental health issues such as depression and anxiety [29–31].

In our study, approximately three-fourths of the physicians had depression symptoms, four out of five had anxiety, and about half exhibited symptoms of stress. Another study in Türkiye reported that 64.7% of physicians had depression, 51.6% had anxiety, and 41.2% experienced stress symptoms [32]. In a study conducted by Elbay et al. during the COVID-19 pandemic in Türkiye, the prevalence of depression was 39.8%, % and that for anxiety was 26.2%, and in a study among primary healthcare workers before the pandemic, the prevalence of depression was 20.7%, anxiety was 27.2% and that for stress was 18.2% [32,33]. According to the results of the Turkey Chronic Diseases and Risk Factors Prevalence Study, the prevalence of depressive disorders (major + minor) in the population was 9% [34]. In a multicenter study conducted by Ain et al. in Malaysia, it was reported that 51.6% of physicians had depression, 51% had anxiety, and 43.9% exhibited stress symptoms [35]. Dave et al. conducted a study in India, and reported a depression prevalence of 27.7%, that for anxiety of 36.6%, and that for stress of 24.2% [36]. These prevalences are higher than those reported in studies conducted in the general population in the same countries [37,38]. Even if the negative emotional effects of the COVID-19 pandemic on healthcare workers have been more pronounced, mental health issues among physicians are more common than in the general population in any case. Among physicians, numerous factors related to the profession, patient demands, administrative and system-related factors, and many other causes converge to become sources of stress. As a result, the emotional problems experienced by physicians can lead to feelings of helplessness, a loss of motivation for work, and questioning the professional decisions they have made, potentially leading to regret [39,40]. Herein, approximately nine out of every ten physicians had some level of regret about their career choice. While there was no relationship between career regret and sociodemographic characteristics, those with fewer years of professional experience had lower regret scores. On the other hand, as the physicians’ levels of career regret increased, their levels of depression, anxiety, and stress also increased. This demonstrates that independent of other factors, physicians’ emotional levels are closely related to their levels of career regret. When chronically exposed to stressors, they are at risk of experiencing physical and psychological burnout. Among physicians, burnout syndrome has become a global problem [41]. Studies have reported physician burnout rates of 54.4% in the United States, 50% in Brazil, 72% in Japan, and 60.6% in China [42–45]. In a study conducted in Türkiye, only 3.1% of physicians reported not experiencing burnout and stated that they enjoyed their work [46]. Burnout reduces physicians’ job satisfaction and can lead to them regretting their career decisions. A study in China by Tian et al. [41] found that 46.6% of physicians would not choose to pursue medicine again if given the chance, and 18.1% were uncertain. Moreover, 83.6% exhibited burnout symptoms, and nearly half of these physicians regretted their career decisions, with only 2.9% being satisfied with their current conditions. The same study found that, similar to the current study, physicians with fewer years of experience experienced less regret, but in contrast to the present findings, those with lower income and those who were married reported higher regret. Family circumstances, income level, having children, and various child-related factors (such as caregiver availability and educational opportunities) can all influence specialty choices in medicine and working conditions following specialization.

In a multicenter study conducted by Eser et al. [47], examining the factors that influence medical students’ desire to leave Türkiye, 39.1% of 9881 medical students reported that they would not choose to attend medical school again if they could turn back time. Furthermore, 70.7% of the students expressed an intention to emigrate from Türkiye, and this intention was associated with the working conditions in the country as a driving factor and the social environment and lifestyle abroad as an attractive factor.

In a recent study conducted in Türkiye, it was reported that 46.3% of 575 medical students intended to migrate, 53.7% planned to live in Türkiye over the long term, and higher levels of depression and stress were significantly associated with migration [48]. It was reported that the number of registration certificates (good-standing) obtained from the Turkish Medical Association regarding whether or not there are any negative records regarding the execution of the professional activities of physicians who are Turkish citizens and want to go abroad increased from 59 in 2012 to 1405 in 2021 [49]. According to data from the Organisation for Economic Co-operation and Development, 8028 foreign physicians migrated to the United States in 2021, of whom 87 were trained in Türkiye. In the same year, it was reported that there were 52,194 foreign-trained physicians in Germany, of whom 1407 were trained in Türkiye^1^.

Therefore, although this study showed the depression, anxiety, stress, and professional decision regret levels of physicians working in Ankara University Hospital, it is thought to be globally important because physicians who receive medical education in Türkiye migrate to work in developed countries. Additionally, from a forensic medical perspective, the high anxiety, depression, stress, and professional regret levels in physicians may have a negative impact on the likelihood of suicidal behaviors and medical malpractice.

## 5. Conclusions

Depression, anxiety, and stress, regardless of sociodemographic characteristics, are common among physicians, and these conditions are associated with their level of professional regret. Therefore, improving working conditions, labor rights, solving economic and other issues, and ensuring the well-being of physicians can help prevent mental health problems. Early screening and rehabilitation for individuals with these issues not only benefit them personally but also improve job satisfaction, strengthen doctor–patient communication, and positively impact healthcare services. On the other hand, ongoing mental health problems can lead to burnout, causing physicians to regret their career choices and potentially seek opportunities in other countries or professions. Ensuring physician employment, a vital part of the healthcare workforce, is crucial for the country’s healthcare system.

## Figures and Tables

**Table 1:** Comparison of the physicians’ DRS scores with their sociodemographic and professional characteristics.

		n (%)	DRS	Statistical Analysis Kw; p
			Median (25th–75th percentile)	
Sex	Female	176 (58.7)	35.0 (25.0–65.0)	1.077; 0.281
	Male	124 (41.3)	40.0 (25.0–65.0)	
Marital status	Married	61 (20.3)	30.0 (25.0–55.0)	2.238; 0.327
	Single	239 (79.7)	40.0 (25.0–70.0)	
Perceived income level	Good	20 (6.7)	40.0 (25.0–65.0)	1.378; 0.502
	Average	186 (62.0)	40.0 (30.0–65.0)	
	Poor	94 (31.3)	35.0 (25.075.0)	
Reason for choosing medical school	Interest in medicine	216 (72.0)	35.0 (25.0–67.5)	0.514; 0.617
	Other	84 (28.0)	40.0 (25.0–65.0)	
Reason for choosing specialty	Interest in specialty	174 (58.0)	35.0 (25.0–65.0)	0.859; 0.391
	Other	126 (42.0)	40.0 (30.0–70.0)	
Future plan	Working in the private sector	61 (20.3)	35.0 (25.0–75.0)	2.689; 0.442
	Private practice	159 (53.0)	40.0 (25.0–65.0)	
	Working abroad	80 (26.7)	37.5 (30.0–65.0)	

**Table 2:** Comparison of the physicians’ age and number of years in the profession with levels of career decision regret.

DRS	Years in the profession		Statistical analysis Kw; p	Age		Statistical analysis Kw; p
	Mean (SD)	Median (25th–75th percentile)		Mean (SD)	Median (25th–75th percentile)	
Not regretful at all for the career choice	2.1 (2.4)	1.0 (1.0–2.0)	12.827; 0.005	27.1 (2.4)	27.0 (26.0–28.0)	0.749; 0.862
Slightly regretful due to the career choice	2.5 (1.8)	2.0 (2.0–3.0)		27.2 (2.0)	27.0 (26.0–28.0)	
Regretful due to the career choice	2.3 (1.1)	2.0 (1.0–3.0)		27.0 (1.3)	27.0 (26.0–28.0)	
Very regretful due to the career choice	2.1 (1.1)	2.0 (1.0–2.0)		26.9 (1.4)	27.0 (26.0–28.0)	

**Table 3:** Comparison of the physicians’ DASS-21 scores with their sociodemographic and professional characteristics.

Characteristics		DASS-21		
		Depression Median (Q1–Q3)	Anxiety Median (Q1–Q3)	Stress Median (Q1–Q3)
Sex	Female	9.0 (4.0–16.0)	9.0 (3.5–16.0)	9.0 (4.0–16.0)
	Male	12.0 (5.0–15.0)	11.5 (5.5–16.0)	11.5 (5.0–16.0)
Test value; p		0.626; 0.531	1.285;0.199	0.665; 0.506
Marital status	Married	8.0 (4.0–14.0)	9.0 (3.0–14.0)	9.0 (3.0–15.0
	Single	11.0 (5.0–16.0)	11.0 (5.0–16.0)	11.0 (5.0–16.0)
Test value; p		1.127; 0.260	1.387; 0.166	1.094; 0.274
Perceived income level	Good	13.0 (4.0–15.5)	11.0 (6.5–13.5)	11.0 (6.5–16.5)
	Average	10.0 (5.0–16.0)	11.0 (4.0–16.0)	10.0 (4.0–16.0)
	Poor	9.0 (3.0–15.0)	1.0 (5.0–16.0)	10.0 (4.0–15.0)
Test value; p		1.565; 0.457	0.003; 0.998	0.928;.0.629
Reason for choosing medical school	Interest in medicine	10.0 (4.0–15.0)	10.0 (3.5–16.0)	11.0 (4.0–15.0)
	Other	10.0 (5.0–16.5)	11.0 (6.0–16.0)	9.0 (4.0–17.0)
Test value; p		1.020; 0.308	1.291; 0.197	0.212; 0.832
Reason for choosing specialty	Interest in specialty	10.0 (4.0–16.0)	10.0 (4.0–16.0)	10.0 (4.0–16.0)
	Other	10.5 (4.0–16.0)	11 (6.0–16.0)	9.5 (4.0–16.0)
Test value; p		0.455;0.649	0.992;. 0.321	0.155;.0.877
Future plan	Working in the private sector	10.0 (4.0–16.0)	10.0 (4.0–14.0)	9.0 (5.0–16.0)
	Private practice	10.0 (4.0–15.0)	9.0 (4.0–16.0)	10.0 (4.0–16.0)
	Working abroad	12.5 (5.0–16.5)	13.0 (5.5–16.0)	11.0 (5.0–15.5)
Test value; p		4.890;.0.180	7.743;.0.052	1.815;.0.612

**Table 4:** DASS-21 scores of the participants.

DASS-21	Score	
	Mean (SD)	Median (Q1–Q3)
Depression	10.2 (6.2)	10.0 (4.0–16.0)
Anxiety	10.2 (6.1)	10.0 (4.3–16.0)
Stress	10.2 (6.2)	10.0 (4.0–16.0)

**Table 5:** Categorical assessment of physicians’ DASS-21 scores.

	Depression n (%)	Anxiety n (%)	Stress n (%)
Normal	79 (26.3)	64 (21.3)	127 (42.3)
Mild	30 (10.0)	24 (8.0)	19 (6.3)
Moderate	44 (14.7)	28 (9.3)	31 (10.3)
Severe	33 (11.0)	25 (8.3)	58 (19.3)
Extremely severe	114 (38.0)	159 (53.0)	65 (21.7)
Total	300 (100.0)	300 (100.0)	300 (100.0)

**Table 6:** Correlation between Scores from DASS-21 and DRS.

	Depression score	Anxiety score	Stress score	DRS
Depression score	-	0.838; 0.000	0.855; 0.000	0.692; 0.000
Anxiety score	0.838; 0.000	-	0.785; 0.000	0.676; 0.000
Stress score	0.855; 0.000	0.785; 0.000	-	0.667; 0.000
DRS	0.692; 0.000	0.676; 0.000	0.667; 0.000	-

## Footnotes

1 Organisation for Economic Co-operation and Development (2022). Health Workforce Migration: Migration of doctors. [online]. Website https://stats.oecd.org/Index.aspx?QueryId=68336# [accessed 23 November 2023].
